# Management of Medically Compromised Prosthodontic Patients

**DOI:** 10.1155/2022/7510578

**Published:** 2022-01-11

**Authors:** Pratistha Ghimire, Pramita Suwal, Bishal Babu Basnet

**Affiliations:** Department of Prosthodontics and Crown-Bridge, College of Dental Surgery, BP Koirala Institute of Health Sciences, Dharan, Nepal

## Abstract

The medical evaluation of patients considering prosthodontic treatment is a vital step in the treatment planning. The prosthodontist should be able to assess the inherent risks associated with the treatment of patients with systemic conditions. Many factors are associated with evaluating the patient's health status and risk including the patient's current and past medical and dental history, current and past use of medications, type of treatment, length of treatment, invasiveness of treatment, and degree of urgency of treatment. In this article, some of the systemic diseases like arthritis, cardiovascular diseases, endocrine disorders, hematologic and oncologic disease, neurologic disorders, bone disorders, pulmonary diseases, liver diseases, and chronic kidney disease that commonly affect aged individuals are reviewed. The prosthodontic considerations that should be taken care of while managing patients with these systemic conditions will also be discussed.

## 1. Introduction

Older adults with chronic medical conditions are increasing in number as dental patients. So, a wide range of knowledge regarding medical conditions and drugs considerations is deemed necessary in dentists. Various chronic disorders and their treatments require alterations in the dental treatment. Serious clinical consequences may result due to failure of appropriate treatment modifications. Thorough evaluation followed by risk assessment should be done to determine whether a planned procedure can be safely implicated for successful dental management of a medically compromised patient while performing dental treatments. Risks and benefits should be examined for a dental treatment, and benefits overweigh the risk of medical complications [1].

In this article, some of the systemic diseases (arthritis, cardiovascular diseases, endocrine disorders, hematologic and oncologic diseases, neurologic disorders, bone disorders, pulmonary diseases, liver diseases, and chronic kidney disease) that commonly affect the aged individuals are reviewed. General considerations of these systemic disorders along with the prosthodontic management modifications needed for providing adequate oral health care will also be discussed.

## 2. Medical Considerations of Patients

### 2.1. Arthritis

The most common form of arthritis is osteoarthritis, which is noninflammatory, whereas the most common form of inflammatory arthritis is rheumatoid arthritis [2–4].

#### 2.1.1. Osteoarthritis

Osteoarthritis is the most common chronic disease in elderly, characterized by progressive pathological change of the hyaline cartilage along with bony joints that subsequently leads to degradation of cartilage or bone [5]. It affects the joints of hands, knees, hips, and spine. It also affects the temporomandibular joints (TMJs) [6].

*(1) Prosthodontic Implications in Osteoarthritis of TMJs.* Due to the difficulty in opening mouth, special impression trays to make impression are often necessary. There is problem in complete denture (CD) construction since the mandibular movements are painful. It is difficult to record and repeat jaw relation records. Because of subsequent changes in the joint, occlusal correction must often be made. During tooth preparation for fixed dental prosthesis (FDP), appointments should be kept short. Longer appointments can be segmented into shorter ones since there is difficulty for opening mouth for a longer period.

In the active phase of the disease, full mouth rehabilitation and fixed dental prosthesis should be avoided and only reversible as well as stabilization procedures can be performed [6].

Regarding the use of implants, there have been no articles published reporting on implant survival in patients with osteoarthritis [7].

#### 2.1.2. Rheumatoid Arthritis

Rheumatoid arthritis (RA) is a chronic inflammatory disease in which synovial inflammation results in destruction of joint tissues. There is self-attack in the immune system and manifests as bilateral synovitis typically affecting joints of hands and feet [8]. It may also affect the TMJs [9, 10].

*(1) Prosthodontic Implications in Rheumatoid Arthritis* [9, 10].

- Dentists should be updated about the drugs being used with their side effects and interactions with other medications
- Because of reduced manual dexterity, patients cannot insert or remove partial denture. So, fixed denture therapy may be suitable.
- Patients with prosthetic joints may require prophylactic antibiotics before surgical procedures like dental implant placement
- There are various problems associated with prosthetic rehabilitation in cases with rheumatoid arthritis of TMJ, which include change in occlusion and difficulty in recording jaw relation. Therefore, unloading appliances or provisional construction before the definitive treatment is beneficial.
- Since disease occurs as transient between acute and chronic phase, definitive treatment should be delayed until the disease is cured

### 2.2. Cardiovascular Diseases

The most common cardiovascular diseases of prosthodontic concern include hypertension, angina, myocardial infarction, infective endocarditis, congestive heart failure, and anticoagulant therapy [10].

For all the patients with cardiovascular diseases, the general stress reduction protocol should be followed [11]:

- Adequate counseling about fears or anxiety of patients
- Shorter morning appointment
- Preoperative sedation: with short acting benzodiazepine one-hour preoperative or the night before appointment.
- Intraoperative sedation (N_2_O-O_2_) may also be considered
- Profound local anesthesia
- Adequate postoperative pain analgesia
- We should inform the patient about the positive response of the treatment on the evening of the procedure

#### 2.2.1. Hypertension

Hypertension (HTN) is an abnormal increase in arterial pressure that may be fatal if sustained and untreated [1]. The ACC/AHA in 2017 gave the classification of blood pressure (Table 1) [12].

Oral manifestations of hypertension result not directly due to HTN itself but occur as a side effect of antihypertensive drugs. They include xerostomia caused by diuretics, lichenoid mucosal lesions, burning mouth, loss of taste sensation (angiotensin-converting enzyme inhibitors), and gingival hyperplasia (calcium channel blockers). Extra-oral manifestation like sialadenosis may also occur [1].

*(1) Prosthodontic Considerations in HTN* [1].

- Stress reduction protocol should be followed for anxious patients
- During treatment, abrupt changes in body position should be discouraged to minimize the risk of orthostatic hypotension
- Although morning appointment with minimal waiting time is preferable, lower blood pressure occurs during the daytime rather than morning. So, afternoon appointments are considered safer
- Local anesthesia with vasoconstrictor should either be avoided or used in low doses in uncontrolled hypertension cases. However, using 1–2 cartridges of 2% lidocaine with 1 : 100000 epinephrine may be advantageous and carries less clinical risks.
- Nonsteroidal anti-inflammatory drugs (NSAIDs) are used only for short therapy
- Sharp edges of dentures should be well trimmed and polished to avoid trauma
- Utmost care should be provided to avoid soft tissue abrasion during denture fabrication
- Artificial salivary lubricants can be advised for better post-therapy results so as to compensate the effect of xerostomia
- To reduce gingival bleeding, supragingival margins are advised
- Use of epinephrine for gingival retraction should be carefully administered. Local hemostasis should not be attained with topical vasoconstrictor.
- Resting systolic pressure >180 or diastolic pressure >110 indicate that all elective procedures should be delayed until blood pressure can be reduced to a safer level

#### 2.2.2. Angina Pectoris

Angina pectoris is defined as the chest pain that is a result due to reduced blood flow to cardiac tissue [1].

Classical symptoms include retrosternal pain that develops during stress or physical exertion, radiating to shoulders, left or right arm, neck, or mandible, lasts for less than 5 minutes, and is promptly relieved with rest or sublingual nitroglycerine tablets [1].

During the dental treatment, angina attack may occur, which can be managed as follows [1]:

- Firstly, discontinue the procedure
- Position the patient semi-upright or upright
- Administer oxygen
- One tablet (0.3–0.6 mg) of nitroglycerin is given sublingually
- If signs or symptoms do not resolve within 2 to 3 min, another dose of nitroglycerin is administered, and be ready to accompany the patient to the emergency
- A third nitroglycerin may be given three minutes after the second one

The chest pain that continues even after 3 tablets of nitroglycerine may be a signal to possibility of myocardial infarction. The patient should be taken to a higher care center immediately.

Canadian Cardiovascular Society has given the classification of angina (Table 2) [13].

*(1) Prosthodontic Management in Angina Pectoris* [4]. Patients with mild angina (up to one attack per month) can undergo most nonsurgical dental procedures with normal protocol. Vital signs should be monitored during procedure. Nitroglycerine is prescribed to the patient. Extensive treatment like implants is postponed or done with nitrous oxide sedation, and only up to 0.004–0.005 mg of adrenaline is used.

Patients with moderate angina (up to one attack per week) are prescribed to take sublingual dose of nitroglycerine prior to extensive treatment such as implant surgery. Adequate anxiolytic treatment with oxygen supplementation is recommended.

Patients with unstable angina (daily episodes) are limited to examination procedures and are advised an absolute contraindication for elective dental surgery like placement of dental implants.

#### 2.2.3. Myocardial Infarction

Myocardial infarction (MI) is defined as prolonged ischemia or hypoxia due to deficiency of blood supply in the coronary artery causing injury to the myocardium [4].

*(1) Dental Management of MI*. Dental management of MI is same as that of angina pectoris. Additional considerations include if the patient is under anticoagulants, the international normalized ratio (INR) should be determined on the day of treatment, and treatment should be provided within the recommended limits, i.e., <3.5, with adequate bleeding control for surgery. Further hemostasis is mandatory in cases with antiplatelet medications.

Implant considerations in patients with MI based on the duration of last MI attack are shown in Table 3 [4].

#### 2.2.4. Infective Endocarditis

Infective endocarditis (IE) is the infection of the heart valves or endothelial surfaces of the heart [4].

*(1) Prosthodontic Management in IE* [4, 14].

(i) Endocarditis prophylaxis is recommended in various dental procedures such as dental implants or subgingival cord placements in many cases, which includes prosthetic heart valves, past infective endocarditis, cyanotic congenital cardiac disease etc.
The newly revised regime for prophylaxis includes the following:
  - 2 gm amoxicillin per oral (PO) 60 minutes prior or
  - For those unable to take oral medication, 2 gm of ampicillin intramuscular (IM) or intravenous (IV) 30 minutes before procedure or
  - If the patient is allergic to penicillin, 600 mg clindamycin or 2 gm cefalexin PO 1 hour before procedure or
  - If patient is allergic to penicillin and unable to take oral medication, 600 mg clindamycin IV or 1.0 gm of cefazolin IM or IV 30 minutes before procedures
(ii) The prophylaxis for endocarditis is not necessary for mere insertion of prosthesis or making impressions
(iii) For the patients with poor oral hygiene potential or with past history of multiple endocarditis episodes, implants therapy may be unsuitable
(iv) In the instances when implants are necessary, endoosseous implants with an adequate width of attached gingiva are preferred

#### 2.2.5. Congestive Heart Failure

Congestive heart failure (CHF) is a pathophysiologic state characterized by abnormality in cardiac function, which causes failure of heart to pump adequate blood to the tissues [1].

Many drugs that are used in management of HF result in dryness of mouth and oral lesions. Digitalis toxicity causes increased gag reflex and hypersalivation. So, as a dentist, we must be able to recognize these signs.

New York Heart Association has classified CHF (Table 4) based on which prosthodontic management is done [15].

*(1) Prosthodontic considerations in CHF* [4]. Stress reduction protocol is indicated for all patients with CHF. NYHA class I and II can receive routine outpatient dental care. Medical consultation is not indicated unless there exist additional systemic diseases. For NYHA III and IV, medical consultation recommended for all complex implant therapies that involve ridge augmentations, sinus grafting and subperiosteal implants with extensive periosteal reflection, full-arch implant therapy, and autogenous block bone augmentations.

#### 2.2.6. Anticoagulant Therapy

Anticoagulant therapy is commonly encountered in cardiac patients. For a healthy person without anticoagulant therapy, normally the value of INR should be 1. An INR between 2 and 3 is recommended for those under anticoagulants. Higher INR values of 2.5–3.5 may be required in high-risk situations (prosthetic heart valves) [4].

Indication for anticoagulation should be identified because sometimes a short period of discontinuation of anticoagulants without substantially increasing the risk of thrombotic events can be done. Anticoagulants should not be discontinued in patients with mechanical valve prostheses. The patient's physician should be consulted when needed. If the patient is in long-term anticoagulant therapy and stably anticoagulated on warfarin, the INR should be checked 72 hours before surgery [4].

*(1) Dental Management for Patients under Anticoagulant Therapy* [1].

- To determine the level of anticoagulation, consultation to physician is always recommended
- No adjustments in oral anticoagulant are indicated, if invasive procedures or minor oral surgery is planned and the INR is between 2 and 3.5
- Use of oxidized cellulose or collagen sponges and sutures may decrease the risk of bleeding in patients who are using oral anticoagulants. Consideration for the use of mouthwash with 5% tranexamic acid four times a day for two days can also be done.
- NSAIDs and COX-2 inhibitors should not be prescribed in patients taking warfarin as analgesia following dental surgery

### 2.3. Endocrine Disorders

#### 2.3.1. Diabetes Mellitus

Diabetes mellitus (DM) is a group of metabolic diseases characterized by increased blood glucose level and inability to produce and/or use insulin.

Diabetes is classified broadly as type 1 and 2 (Table 5) [1].

A level of fasting blood sugar ≥126 mg/dl and postprandial blood sugar ≥200 mg/dl is considered as diabetes according to American Diabetic Association [16]. Symptoms of diabetes include polyuria, polydipsia, polyphagia, weight loss, and visual disturbances. Complications may be short term like hypoglycemia and diabetic ketoacidosis and long term like diabetic retinopathy, diabetic neuropathy, and diabetic nephropathy. Periodontitis is considered as the sixth complication of DM.

Oral manifestations in uncontrolled DM include xerostomia, periodontitis, burning mouth syndrome, delayed wound healing, alveolar bone resorption, and candidiasis [16].

Usually dental therapy is deferred in cases with uncontrolled DM. If suspected, physician consultation is recommended. Low blood sugar reaction is best avoided by checking with a glucometer prior to the procedure.

Diabetic patients may develop hypoglycemia while in dental chair. Emergency management of hypoglycemia should be done immediately [1, 4, 17]:

- The blood glucose level of the patient should be checked with a glucometer
- If the patient is conscious, 15 gram of carbohydrate (3–4 teaspoon sugar, 4–6 ounce fruit juice or soda, a hard candy, or small amount of honey/sweet syrup) is given orally
- If the patient is unconscious, IV 50 ml of 50% dextrose solution or 1 mg of glucagon IV/IM is given
- Usually the symptoms resolve in 10–15 minutes. But the patient should be monitored for 30–60 minutes after recovery and the glucometer confirmation of normal blood sugar level is done before the patient leaves.

In the postoperative period, eating the right diet is considered a critical part of diabetic therapy. Postoperative diet plan should be planned with consultation with physician and dietician, and maintenance of nutrients is confirmed.

Occasionally, diabetic patients may go into ketoacidosis (diabetic ketoacidosis), the symptoms of which include increased thirst, dehydration, fruity smell of breath, severe breathing, and shortness of breath. If these signs or symptoms appear, emergency management should be done immediately [17]. Management of diabetic ketoacidosis is done by providing adequate hydration, insulin, and electrolyte replacement.

*(1) Prosthodontic Considerations in DM* [4, 18–20]:

1. Take proper medical history of the patient
2. Establish levels of glycemic control early in the treatment process and query about the diet taken by the patient
3. Scheduling the patient's visit preferably in the morning
4. Stress reduction protocol should be followed
5. Oral hygiene instructions, regular prophylaxis, and monitoring of periodontal health is advised
6. Use of antibiotics is recommended in case of infection
7. It is recommended to avoid NSAIDs if the patient is on sulfonylureas

  In RPD:
(i) Maintenance of good oral hygiene must be accomplished first
(ii) All components of RPD should be well adapted to the underlying tissues
(iii) Utmost oral and prosthesis care instructions should be delivered

  In CD:
(i) Always use tissue friendly material
(ii) Mucostatic impression technique is suggested
(iii) Neutral zone technique is advised
(iv) Denture flanges should be smooth and polished
(v) Proper oral hygiene instructions should be given along with regular follow-up visits
(vi) If patient has less salivation, proper therapy for maintenance of wet environment (e.g. water sipping, sugarless gum)
(vii) Frequent evaluation of denture is necessary

  In FDP:
(i) Avoid traumatization of soft tissues during tooth preparation
(ii) Supragingival finish line is better
(iii) Chamfer margin on the facial aspect is considered a better option because it causes minimal
(iv) Group function or mutually protected occlusal scheme is regarded as better choice for periodontally compromised teeth
(v) Proper flossing is advised to maintain the oral hygiene
(vi) Hygienic pontic is preferred for the ease of cleansing

  In implants or implant-supported denture:

- Surgical procedure is started only after adequate control of diabetic state
- Pre and post implant surgery, antimicrobial cover is recommended
- Smoking cessation, proper oral hygiene, and antiseptic mouth rinses is recommended
- Glucose level should be monitored even after implant placement
- Implant dentistry is not contraindicated in most diabetic patients; however, their medical care should be controlled

According to the level of postprandial blood glucose, plan for implant procedures is presented in Table 6 [4].

#### 2.3.2. Thyroid Disorders

Disorders in thyroid can either lead to overfunctioning (hyperthyroidism) or underfunctioning (hypothyroidism) of the thyroid glands. Patients with poorly controlled thyroid disorders can experience complications [1].

Patients with hyperthyroidism are predisposed to [1]

- Adverse interaction with epinephrine
- Life-threatening cardiac arrhythmias
- CHF
- Thyrotoxic crisis (thyroid storm, precipitated by infection or surgical procedures)

Complications that may occur in patients with hypothyroidism [1]:

- Exaggerated response to CNS depressants (sedatives and narcotic analgesics)
- Myxedematous coma (precipitated by CNS depressants, infection, or surgical procedures)

For well-managed thyrotoxic patients with thyroid disease, normal concentration of vasoconstrictors can be used, but the use of epinephrine is avoided in untreated or poorly managed thyrotoxic patients [1].

*(1) Dental Implant Management in Thyroid Disorders* [10].

- The most common thyroid disorder patients seen in implant dentistry are the ones with known and treated thyroid disease, i.e., without any symptom are considered as low risk and implant surgery can be done safely.
- Patients with thyroid disorder who have no symptoms are considered as moderate risk. These patients are managed with normal protocol along with stress reduction protocol. Use of epinephrine should be limited in moderate to advanced implant procedures.
- Patients with symptoms are considered as high risk. Such patients should have only examination procedures performed, and all other treatment should be defaced until control of the condition after adequate medical or laboratory confirmation.

#### 2.3.3. Adrenal Gland Disorder

- Disorders of the adrenal glands can result either in underproduction (hypoadrenalism) or overproduction (hyperadrenalism) of adrenal products. Both of these disorders may face problems due to stress and during dental treatment [1].
- Patients with high risk of adrenal suppression are those who have received a dose of 20 mg or more of cortisone or equivalent for 2 weeks or more within two years of dental treatment
- Patients with moderate risk of adrenal suppression are those formerly on steroid therapy for longer than seven days within a year of dental treatment
- Patients with low risk of adrenal suppression are in alternate dose of steroid therapy, which ended one year or more before the implant procedure [4]

*(1) Prosthodontic Considerations in Case of Adrenal Gland Disorders.*

- In high-risk patients, for implant surgery, steroid dose should be doubled the day before surgery and maintenance dose is returned to normal, the day after the surgery
- In moderate-risk patients, for implant surgery, the dose is doubled on the day of surgery. After surgery, on each day, the dose in reduced to 50% for a period of three days. General anesthesia may be used to reduce anxiety in the apprehensive patient.
- In low-risk patients, dental procedures are scheduled on the same day steroids are taken by the patient. On the second day, the dose is reduced to 50%. Then, on the third day, alternate day schedule is resumed. In addition, sedation and antibiotics are used [4].

### 2.4. Hematologic Disorders

Hematologic disease of concern to prosthodontists includes the following [4]:

- Disorders of red blood cells—polycythemia, anemia
- Disorders of white blood cells—leukocytosis, leukopenia
- Disorders of platelets—thrombocytopenia

*General Prosthodontic Considerations in Hematologic Disorders*. There is no risk while performing removable prosthodontic treatments; however, trauma to the tissues should be minimized in postinsertion adjustments. Handling the oral tissues delicately in all the steps is beneficial to reduce chances of ecchymosis.

#### 2.4.1. Polycythemia

In case of polycythemia, dental implants are contraindicated [4].

#### 2.4.2. Dental Implant Considerations in Anemia

In case of long-term anemic patients, bone maturation and development are often impaired. There is reduction in 25%–40% trabecular pattern [4]. So, the initial character of the bone needed to support the implant is significantly affected and time needed for proper interface formation is longer. In anemic patients, abnormal bleeding causes difficulty in placement of subperiosteal implants. The risk of postoperative infection increases due to increased edema, which can affect the long-term maintenance of implants or abutment tooth. In majority of patients with anemia, implant procedures can safely be carried out, but the minimum baseline recommended is 10 mg/dl especially for implant surgery. However, antibiotic cover is recommended before and after the surgery [4].

#### 2.4.3. Dental Implant Considerations in Leukopenia and Leukocytosis

Most common complications that can compromise the success of implant are infection and delayed healing. These may further increase the risk of secondary infection [4].

#### 2.4.4. Dental Implant in Thrombocytopenia

Dental implant in thrombocytopenia is contraindicated if the platelets count is <50,000 U/L [21].

### 2.5. Oncologic Disease

More than 90% cases of oral cancer are squamous cell carcinoma, which is most commonly managed medically by surgery and radiation therapy [1].

#### 2.5.1. Prosthodontic Considerations in Cancer

(I) Before commencement of cancer therapy, oral cavity evaluation is recommended in all cases [1]:
  - Edentulous regions should be surveyed
  - Impressions may be taken for surgical obturators
  - Maintenance of oral health during cancer therapy
(II) If head and neck radiation therapy (RT) are scheduled, it is recommended that [22, 23]
  - For any oral surgical procedures, adequate healing time is provided before induction of radiotherapy
  - Temporary restoration can be placed, while cosmetic and prosthodontic treatments can be delayed when the time is limited
  - Complications of RT include mucositis, taste alteration, xerostomia, trismus, opportunistic infections, radiation caries, and osteoradionecrosis
(III) During cancer therapy, other surgical interventions (extraction, maxillary prosthetic obturator, primary implant placement, and preprosthetic procedures) may be carried out
(IV) Postcancer treatment management includes the following [22, 24, 25]:

- Avoid wearing dentures during first six months after completion of radiotherapy
- Patients are instructed to return to the dentist if any sore spots develop
- Ill-fitting dentures should be replaced by new dentures
- In case of severe chronic xerostomia, it is advised to apply petrolatum to the mucosal surface of the denture for adhesion
- 12–18 months after radiation therapy, implants can be placed, but it requires knowledge of tissue irradiation fields, degree of healing, and vascularity of the region. For example, implants placed in the posterior mandible pose higher risk than those in the maxilla or anterior mandible.

A five-year survival rate of 90% for oral implants has been reported in patients who have undergone surgery and radiotherapy for oral cancer [26].

### 2.6. Osteoradionecrosis

Osteoradionecrosis of jaw results from failure to heal after high dose of irradiation. This condition is due to hypocellularity, hypovascularity, and hypoxia of bone tissues that are induced by radiation. Soft tissue necrosis often presents preceding bone involvement at diagnosis. There is higher risk of osteoradionecrosis in the mandible than in maxilla [22, 24, 25].

Protocols for reducing the risk of osteoradionecrosis are as follows [22, 24, 25]:

- Prefer endodontic treatment over extraction
- Use of LA with no or low concentration of epinephrine
- Atraumatic surgical technique
- Prophylactic antibiotics and antibiotics during healing week
- Hyperbaric oxygen therapy before invasive procedures

If bony necrosis has already occurred, first conservative management is done [22, 24, 27]:

- Irrigation with saline or antibiotic solution is done to exposed bone
- Bony sequestrum is removed
- Broad-spectrum antibiotics (ampicillin, amoxicillin/clavulanic acid, doxycycline) are prescribed, if swelling and suppuration are present
- If the response to conservative management fails, surgical resection of bone is done

### 2.7. Bone Disorders

#### 2.7.1. Osteoporosis

Osteoporosis is defined by low bone mass, increased microstructural deterioration, and bone fragility [4].

*(1) Prosthodontic Management in Osteoporosis* [28].

- Preservation of underlying tissue structure should be attained with proper design of complete denture
- Mucostatic or open mouth impression technique is recommended
- Use of non- or semianatomic acrylic teeth with narrow buccolingual width is advised
- Extended tissue rest from dentures is advised for at least 10 hours per day
- Optimal use of soft liners may be considered
- Frequent relining of dentures is often required

*(2) Dental Implant Management in Osteoporosis* [4].

- For bone volume and density, osteoporosis is a significant factor; however, it does not contradict the placement of implant. The design of implant should be such that it provides greater bone contact and density (large width, long length, hydroxyapatite coating, etc.).
- In addition, patients are advised to have adequate dietary calcium intake and healthy lifestyle

#### 2.7.2. Osteitis Deformans (Paget's Disease)

Osteitis deformans (Paget's disease) is a common metabolic disease characterized by slow, progressive, uncontrolled resorption and deposition of bone [4]. The maxilla and mandible are affected in 20% of cases [10]. The maxilla is two‐fold involved in comparison with the mandible [4].

*(1) Oral Manifestations of Osteitis Deformans* [29, 30].

- Bilaterally symmetrical bone swelling
- Headache, blindness, deafness
- Difficulty in wearing old dentures
- Formation of gap between teeth (diastema), tooth mobility, malocclusion
- Pathologic fractures
- Leontiasis ossea—when facial bone is affected

*(2) Prosthodontic Management of Osteitis Deformans.*

- Supporting area such as maxillary tuberosities may have continuous enlargement so adjusting dentures frequently may be necessary [10, 31]
- Oral implants are contraindicated in the affected regions [4]

#### 2.7.3. Fibrous Dysplasia

In this condition, replacement of normal bone tissues occurs by fibrous connective tissue in an unorganized fashion [4].

*(1) Oral Manifestations of Fibrous Dysplasia* [4].

- The maxilla is affected twice than the mandible
- Monostotic fibrous dysplasia manifests as a painless, progressive lesion
- Teeth become mobile as a result of disease progression
- Bone may be predisposed to fracture
- Delayed healing and higher incidence of infection is noted

*(2) Implant Consideration in Fibrous Dysplasia* [4].

- In an active lesion area, it is absolutely contraindicated
- In nonlesion areas, it is relatively contraindicated

### 2.8. Neurologic Disorders

#### 2.8.1. Parkinson's Disease

Parkinson's disease is a neurodegenerative disorder characterized by tremors, rigidity, bradykinesia, and postural instability. It is caused due to depletion of neurotransmitters—dopamine [1, 32].

*(1) Prosthodontic Considerations in Parkinson's Disease* [1, 10].

(i) Patient movement in dental set up may be affected by tremor and rigidity.
(ii) There should be maximal pharmacological effect of drugs while working.
(iii) A semireclined position is suitable for those who have difficulty in controlling salivation.
(iv) Proper adjustment of dental chair is done slowly, and the patient is given enough time to be upright sitting position then only rise slowly from dental chair.
(v) There may be chances of orthostatic hypotension, and therefore care should be taken.
CD [33–36]:
  - If the denture is to be provided for the first time, consideration should be given for implants or implant-supported overdentures.
  - Impressions should be made with fast setting impression materials.
  - Denture retention, stability, and support are compromised due to tremors and rigidity of orofacial musculatures as well as drooling of saliva.
  - Neutral zone technique and flange technique are advised for making final impressions.
  - Selective grinding should be done to eliminate any interferences and to gain maximum stability and retention of dentures.
  - Monoplane teeth can be used to establish stable occlusion.
  - Duplication of familiar old denture helps to retain learned muscular control in case where replacement is needed. Older dentures should be replaced because there is high resorptive bone in prolonged denture wearers. In doing so, the previous denture may act as impression tray.
  - Low viscosity polyvinyl siloxane is preferred.
  - Weakness of buccinator muscle alters the dynamics of eating, resulting in food stagnation in the fornix area. The outer surface of the denture is modified to improve its retention (in the lower part) as well as to decrease quantity of food between the buccinator muscle and the prosthetic modification.
RPD [33–35]:
  - Major connectors should not be of smaller dimension.
  - Retainers designed must be retentive.
  - Precision attachments must be avoided.
  - Use of flexible dentures is a good option
FDP [35, 37, 38]:
  - Supragingival or equigingival margins are indicated.
  - Gingival retraction material (an expanding vinyl polysiloxane) for retraction of gingival sulcus.
  - Full-coverage restorations are treatment of choice
  - Resin cements are recommended.
Implant surgery [23, 32, 33]:
  - LA containing epinephrine is used cautiously because if it agonizes with levodopa or entacapone, it will shoot up BP and heart rate.
  - Epinephrine <0.05 mg is considered safe.

There is a marked improvement in the oral and general health using implant-supported prosthesis and its association with increased masticatory ability in patients with Parkinson's disease [37].

### 2.9. Pulmonary Diseases

General precautions that must be followed while managing patients with pulmonary diseases are as follows [1, 4, 39]:

- Take proper history of the patient
- Patient's physician should be consulted for medications and status of the diseases
- Provide stress-free environment during each visit
- Position the patient in upright position
- Avoid drugs causing respiratory depression like narcotics and sedatives
- Avoid bilateral mandibular block anesthesia
- Avoid ultrasonic instrumentation
- Prosthodontic procedures should not be done until emergency, i.e., in case of active fungal or bacterial respiratory disease
- Use of vasoconstrictors and gingival retraction cord not advised

#### 2.9.1. Additional Considerations for COPD

In addition to the general precautions, additional considerations for chronic obstructive pulmonary diseases (COPD) are as follows [1, 4]:

- If the patient is under systemic corticosteroids, supplementation dose is required for major surgical procedures because of adrenal suppression
- Avoid the use of macrolide antibiotics in the patient taking theophylline

#### 2.9.2. Considerations for Asthma

In addition to the general precautions, additional considerations for asthma are as follows [1, 4, 18]:

- Schedule late morning appointment
- The patient's inhaler should be available during each visit
- Aspirin, NSAIDs, and barbiturates are avoided
- LA without epinephrine/levonordefrin is advised in moderate to severe disease
- Prophylactic inhalation of bronchodilators is advised at the beginning of appointment to prevent asthma attack
- Optimal curing of acrylic prostheses is recommended, and material free of methyl methacrylate is preferred
- If acute asthma attack occurs, a short-acting *β*2 adrenergic agonist inhaler is recommended

#### 2.9.3. Considerations for Tuberculosis

In addition to the general precautions, additional considerations for tuberculosis are as follows [1, 32]:

- Only emergency care should be provided to the patient with tuberculosis
- Physician should be consulted for the result of sputum cultures for *Mycobacterium tuberculosis*
- When the results are negative, the patient may be treated normally
- When the results are positive, we have to know that adequate treatment of tuberculosis requires a minimum of 18 months with a post-treatment follow-up
- Standard precautions for infection control are mandatory

### 2.10. Liver Diseases

In patients with liver disease, synthesis of clotting factors and ability to detoxify drugs are most commonly affected. For general management of these patients, strict attention to hemostasis is indicated [4].

#### 2.10.1. Dental Implant Management in Case of Liver Diseases

- Patients with no abnormal lab values for complete blood count, partial thromboplastin time (PTT), and prothrombin time (PT) are under low risk. Normal protocol is indicated for all nonsurgical and simple surgical procedures in these cases.
- Patients with elevated PT less than 1.5 times the control value or slightly affected bilirubin fall under moderate risk. Physician consultation should be done in such cases. Bleeding should be controlled adequately.
- If elevated PT is more than 1.5 times the control value, they fall under high risk for which elective dental procedures are contraindicated [4].

### 2.11. Chronic Kidney Disease

Symptomatic kidney cases without diagnosis should be referred to physicians. Pretreatment evaluation for bleeding disorders should be done, if invasive procedures are planned. If the glomerular filtration rate is <50 ml/min., elective dental care should be postponed until the patient is medically stable and consultation is obtained [1].

### 2.12. Dialysis

For patients receiving peritoneal dialysis, there is no problem with dental management, but those receiving hemodialysis are susceptible to infection. Dental treatment should be provided on the day after hemodialysis. Major surgical procedures should be performed on the day after the end of the week of hemodialysis. If dental treatment is necessary on the day of hemodialysis, protamine sulfate is administered by the physician to block anticoagulant effects of heparin [1].

## 3. Conclusion

The successful management of patient begins from taking the adequate medical history to making the proper treatment plan. Many of the aged patients are already diagnosed with some medical condition before they present for prosthodontic treatment. The prosthodontic procedures are delayed until the medical conditions are evaluated. The drugs taken by the patient for their systemic condition should be known since they have an impact on treatment outcome along with the drug interactions. Medical consultation should always be considered for appropriate treatment modifications, wherever required. Systemic evaluation, as well as the physician, consultation should be an integral part of prosthodontic treatment plan.

## Figures and Tables

**Table 1:** Classification of blood pressure (BP) as per ACC/AHA 2017 [12].

BP classification	Systolic BP	Diastolic BP
Normal	<120 mm Hg	<80 mm Hg
Elevated	120–129 mm Hg	<80 mm Hg
Stage 1 hypertension	130–139 mm Hg	80–89 mm Hg
Stage 2 hypertension	≥140 mm Hg	≥90 mm Hg

**Table 2:** Canadian Cardiovascular Society's classification of angina [13].

Class	Characteristics
Class I	No angina with ordinary activity. Angina with strenuous activity.
Class II	Angina during normal activity (walking up hills, walking rapidly upstairs), with mild limitation of activities.
Class III	Angina with low levels of activity (walking 50–100 yards on the flat, walking up one flight of stairs), with marked restriction of activities.
Class IV	Angina at rest or with any level of exercise.

**Table 3:** Implant considerations in patients with MI based on the duration of last MI attack [4].

Risk	Duration	Implant procedures
Mild	>12 months	Hospitalization if general anesthesia required
Moderate	6–12 months	Postponement of procedure
Severe	<6 months	Postponement of procedure

**Table 4:** New York Heart Association's classification of CHF [15].

Class I	No symptoms and no limitation in ordinary physical activity.
Class II	Mild symptoms and slight limitation during ordinary activity. Comfortable at rest.
Class III	Marked limitation in activity caused by symptoms, even during less than ordinary activity. Comfortable only at rest.
Class IV	Severe limitations. Experiences symptoms even during rest.

**Table 5:** Classification of diabetes [1].

Type 1 (insulin-dependent DM)	(i) *β* cell destruction with lack of insulin
Type 2 (non-insulin-dependent DM)	(i) Insulin resistance and relative insulin deficiency
Gestational	(i) Abnormal glucose tolerance during pregnancy diabetes
Others	(i) Impaired fasting glucose (impaired glucose tolerance)
	(ii) Abnormalities of fasting glucose (abnormal glucose tolerance)
	(iii) Genetic defects of beta cell function, endocrinopathies, drug-induced, etc.

**Table 6:** Implant procedure plan according to postprandial blood glucose level [4].

Risk	Blood sugar level	Implant procedures
Low	<140 mg/dl	Stress reduction protocol, maintain glucose level

Low/medium	140–180 mg/dl	Stress reduction protocol, maintain glucose level Patients with neuropathy, nephropathy, peripheral vascular disease, history of coronary disease, or may be at higher risk. Medical consultation may be appropriate (relative contraindication)

Medium high	180–215 mg/dl	Patients without any secondary manifestations, medical consult may be obtained (relative) Patients with coronary disease or other diabetic-related conditions require medical consult (relative/absolute)

High risk	>215 mg/dl	Medical referral and better glycemic control (absolute contraindication)

## Data Availability

No data were used to support this study.
